# Does laparoscopic hysterectomy + bilateral salpingectomy decrease the ovarian reserve more than total abdominal hysterectomy? A cohort study, measuring anti-Müllerian hormone before and after surgery

**DOI:** 10.1186/s12905-021-01472-5

**Published:** 2021-09-11

**Authors:** Zohreh Tavana, Elham Askary, Tahereh Poordast, Maryam Soltani, Farideh Vaziri

**Affiliations:** 1 Department of OB/GYN, School of Medicine, Shiraz University of Medical Sciences, Shiraz, Iran; 2 Infertility Research Center, Shiraz University of Medical Sciences, Shiraz, Iran; 3 Community Based Psychiatric Care Research Center, Shiraz University of Medical Sciences, Shiraz, Iran; 4 Department of Midwifery, Nursing and Midwifery School, Shiraz University of Medical Sciences, Shiraz, Iran; 5 Department Of Obstetrics and Gynecology, Shahid Faghihi Hospital, Zand Street, 7134846114 Shiraz, , Iran

**Keywords:** Anti-Mullerian hormone, Hysterectomy, Laparoscopy, Laparotomy, Ovarian reserve

## Abstract

**Background:**

Decreased ovarian function and reserve is one of the complications of hysterectomy. In this study, we aimed to compare anti-müllerian hormone (AMH) levels between total abdominal hysterectomy (TAH), and total laparoscopic hysterectomy (TLH).

**Methods:**

In this prospective cohort study, serum levels of AMH were compared between the groups undergoing TAH + bilateral salpingectiomy and TLH, in 66 patients (33 in each group) who referred to the hospitals of Shiraz University of Medical Sciences for hysterectomy during one years of work. The collected information included age, weight, gravidity, parity, regularity of menstrual cycle, uterine weight, blood loss during surgery, and serum levels of AMH before and 6 months after surgery, compared between groups.

**Results:**

Most patients (88% in TAH and 73% in TLH group) aged 40–50 years. Mean age, weight, parity of patients was similar in both groups, while blood loss was significantly less in TLH group (P < 0.01). Median (IQR) of pre-surgical AMH values were 0.40 (0.55) ng/ml in the TLH group and 0.92 (1.23) ng/ml in the TAH group (P = 0.12) that decreased to 0.29 (0.44) ng/ml in the TLH group and 0.15 (0.31) ng/ml in the TAH group (P = 0.02). Also Median (IQR) of the difference between pre and post-surgical AMH values were 0.12 (0.31) and 0.58 (1.17) in TLH and TAH group, respectively (P = 0.003).

**Conclusion:**

The serum levels of AMH decreased significantly after both methods of hysterectomy (laparoscopy and laparotomy), while this decrease was greater in TAH group that shows.

## Background

Hysterectomy is the most common gynecological surgery and the second most common surgery in women, after cesarean section [1, 2]. It is used for a wide range of gynecological symptoms and diseases, such as abnormal uterine bleeding, pelvic pain, and myomatous uterus [3, 4]. According to the Risk Factor Surveillance Survey, 40% of American women undergo hysterectomy during their lifetime [5, 6].

Different techniques are used for hysterectomy, including total abdominal hysterectomy (TAH), vaginal hysterectomy, and total laparoscopic hysterectomy (TLH) [7]. Laparoscopic procedures are suggested to have a shorter length of hospital stay, but increased hospital costs, and duration of surgery [8]. Some studies also suggest higher intra-operative complications, less postoperative pain, and higher quality of life in laparoscopic methods [9], while other studies report no differences in the major complications [8, 10], quality of life and sexual function among different techniques of hysterectomy [11].

Another important issue in hysterectomy is redirected towards the ovaries. As most women undergoing hysterectomy are women of premenopausal age (40–44 years) [6], there are multiple risks and benefits to retaining or removing ovaries during hysterectomy [12]; some suggest retaining at least one ovary, because of the benefits of estrogen production [13], while others suggest that bilateral oophorectomy at the time of hysterectomy, due to decreased risk of breast, ovarian, and total cancers in long-term follow-up [14]. Thus, recent guidelines, such as American College of Obstetricians and Gynecologists (ACOG), suggest retaining ovaries in premenopausal women with negative genetic risk of ovarian cancer and oophorectomy for older women [15]. Yet, review studies suggest inadequacy of evidence in this regard [16].

Even after keeping the ovaries, the risk of ovarian failure is nearly 15% in women undergoing hysterectomy, which is two-folds the women with normal uterus [13], however, in a study, changes in ovarian reserve after hysterectomy have only been attributed to age [17]. Also, the technique of hysterectomy may cause a difference in ovarian function after hysterectomy [18]. Studies have suggested decreased ovarian reserve after laparoscopic hysterectomy [19], as well as abdominal hysterectomy [20], measured by anti-müllerian hormone (AMH), follicle-stimulating hormone (FSH), and estradiol (E2) [21, 22]. Since maintaining ovarian health plays an important role in increasing the health of women, it is preferable to choose a method for maximum ovarian function during hysterectomy.Yet, the pure difference in ovarian reserve among different techniques of hysterectomy have to be further elucidated, in order to be able to choose the most appropriate method for each patient, especially younger women. Thus, we aimed to compare AMH levels before and after surgery between TLH + bilateral salpingectomy and TAH, as the most commonly used techniques.

## Methods

### Study design

In this prospective cohort study, approved by the Ethics Committee of Shiraz University of Medical Sciences (code: 930-01-01-7579), patients who referred to Hazrat-Zeinab and Ghadir Hospitals, affiliated to Shiraz University of Medical Sciences for hysterectomy during one year were recruited. The serum levels of AMH were compared before hysterectomy and 4 months after that between the groups undergoing TAH + bilateral salpingectomy and TLH + bilateral salpingectomy.

The sample size was calculated to be 31, based on previous studies and considering error of 5%, power of 80%, and effect size of 50%, using the following formula:

$$n = \frac{{2(z_{1 - \alpha /2} + z_{1 - \beta } )}\delta^{2}}{{(\mu_{2} - \mu_{1} )^{2} }} + 1$$

Consequently, 33 patients were considered in each group, considering 5% lost to follow-up cases.

After explanation of the objectives and steps of the study to all patients (of similar race), written informed consent was obtained from participants and those who met the inclusion criteria entered the study by convenient sampling method. The inclusion criteria consisted of women who referred to the hospitals of Shiraz university of Medical Sciences with abnormal uterine bleeding (AUB) without anatomical (according to PALM-COIN classification) or hormonal reasons (except FIGO 7 leiomyoma) with uterine size less than 12 weeks, weight less than 600 g [23, 24], and have not responded to medical treatment were scheduled for abdominal or laparoscopic hysterectomy who had not taken any hormonal medication for at least two months before surgery.

The included patients should have had no menopause, and no history of endometriosis, or previous ovarian surgery. The exclusion criteria included the following: all medical comorbidity which lead to decrease ovarian reserve before and after operation including: all women who received gonadotoxic treatment before and after surgery, menopausal women, polycystic ovarian disease, patients who had history of endometriosis, or previous ovarian surgery, patient with auto immune disease, such as: systemic lupus erythematosus, Rheumatoid arthritis, thyroid disease, and also patients with insulin dependent diabetes mellitus or cardiovascular disease.

If the ovaries were (completely or partially) removed during hysterectomy (or had any other adnexal surgery) for any reason, the patient was excluded from the study. The included patients were matched for age, parity, and uterine size and weight, as well as AMH levels. All participants were assigned into two groups of abdominal and laparoscopic hysterectomy, based on patient’s conditions, and preference, as well as surgical principles.

Total abdominal hysterectomy + bilateral salpingectomy was performed by one expert gynecologist according to the standard protocol that included ligation and cutting round and uretero-ovarian ligaments, separation of bladder and cutting uterine artery and cardinal ligament, cutting and suturing vaginal cuff without using electro surgery techniques.

Total laparoscopic hysterectomy + bilateral salpingectomy was performed also by the same gynecologist. In this process, round and uretero-ovarian ligaments were sealed and cut by Ligature 10 (Covedian 1037 with blunt tip as a bipolar vessel sealing device, used with force trial generator to provide a permanent fusion for the vessels up to 7 mm diameter and heat spread is depend on the tip and the duration of activation), then uterine artery was sealed (cutting mood, power 40 W) and cut by bipolar cautery (Günter Bissinger Medizintechnik 's Powergrip bipolar), and bladder was separated by sharp dissection. Finally, vaginal cuff was cut by monopolar cautery and sutured. During both procedures, adhesion of bowel and omentum to anterior wall of abdomen, if present, were released.

For each patient, demographic characteristics of the patient, including age, and weight, as well as obstetrics and gynecologic characteristics, including gravidity, parity and surgical details, including uterine weight, and blood loss during surgery, in addition to the serum levels of AMH before and after surgery were recorded and compared between the two groups.

A venous blood sample was taken from all patients from their left cubital vein in sitting position. The samples’ serum were then separated, kept at − 20 °C and sent to the laboratory, where AMH levels were measured by Backmann kit.

### Statistical analysis

For statistical analysis, collected data were entered to SPSS statistics 15.0 for windows (SPSS Inc., Chicago, IL) and the results were analyzed through descriptive analysis, including frequency, mean and standard deviation (SD) or median (IQR), analytic analysis, including Wilcoxon test to compare the pre and post-surgical values and Mann–Whitney to compare the two groups. As testing the normal distribution of data by Kolmogorov–Smirnov test was statistically significant, non-parametric statistical tests have been used. In this study, a significant level of 0.05 was considered for data analysis.

## Results

The mean ± SD age of the study population was 44.8 ± 4.2 (range 35–50) years. Most patients (88% in TAH and 73% in TLH group) were in the 40–50 year-old age category. The baseline and surgical characteristics of patients were similar in both groups, including mean age, weight (all patients weighted less than 80 kg), parity, uterine weight and baseline AMH levels (P > 0.05), while blood loss was significantly less in TLH group (P < 0.01) (Table 1).

**Table 1:** Demographic characteristics and surgical details of the studied patients in two groups

Characteristic	TLH group	TAH group	P-value
	(n = 33)	(n = 33)	
Age (year)	[38,52]	[35,52]	0.53*
	45.15 ± 3.79	44.57 ± 3.63	
Weight (kg)	[58,80]	[62,80]	0.65*
	69.06 ± 6.04	69.63 ± 4.01	
Parity	[1, 6]	[1, 7]	0.63**
	3(1)	2(1)	
Blood loss (ml)	[103,128]	[275,336]	< 0.01*
	114.5 ± 12.3	304.2 ± 28.8	
Uterine weight (gr)	[338,573]	[354,626]	0.48*
	453.72 ± 118.22	488.34 ± 135.79	

^*^Independent sample T-test (mean ± SD)

^**^Mann–Whitney test (median (IQR))

[Minimum, Maximum]

Median (IQR) of pre-surgical AMH values were 0.40 (0.55) ng/ml in the TLH group and 0.92 (1.23) ng/ml in the TAH group (P = 0.12) that decreased to 0.29 (0.44) ng/ml in the TLH group and 0.15 (0.31) ng/ml in the TAH group (P = 0.02, Table 2). Also Median (IQR) of the difference between pre and post-surgical AMH values were 0.12 (0.31) and 0.58 (1.17) in TLH and TAH group, respectively, which was statistically significant (P = 0.003).

**Table 2:** Association AMH levels between and within the study groups

	TLH group	TAH group	P-value
	(n = 33)	(n = 33)	
Baseline AMH* (ngr/ml)	[0.18,2.60]	[0.07,3.52]	0.12^†^
	0.40 (0.55)	0.92 (1.23)	
Postsurgical AMH (ngr/ml)	[0.06,1.81]	[0.01,1.03]	0.02^†^
	0.29 (0.44)	0.15 (0.31)	
Difference between baseline and postsurgical	[− 0.81,2.43]	[− 0.96,3.45]	0.003^†^
	0.12 (0.31)	0.58 (1.17)	
P-value (pre- vs. post-surgical values)	0.001^*^	< 0.001^*^	

^*^AMH: anti-müllerian hormone

^†^Mann–Whitney test

^*^Wilcoxon test

[Minimum, Maximum]

Median (IQR)

**Fig. 1:**
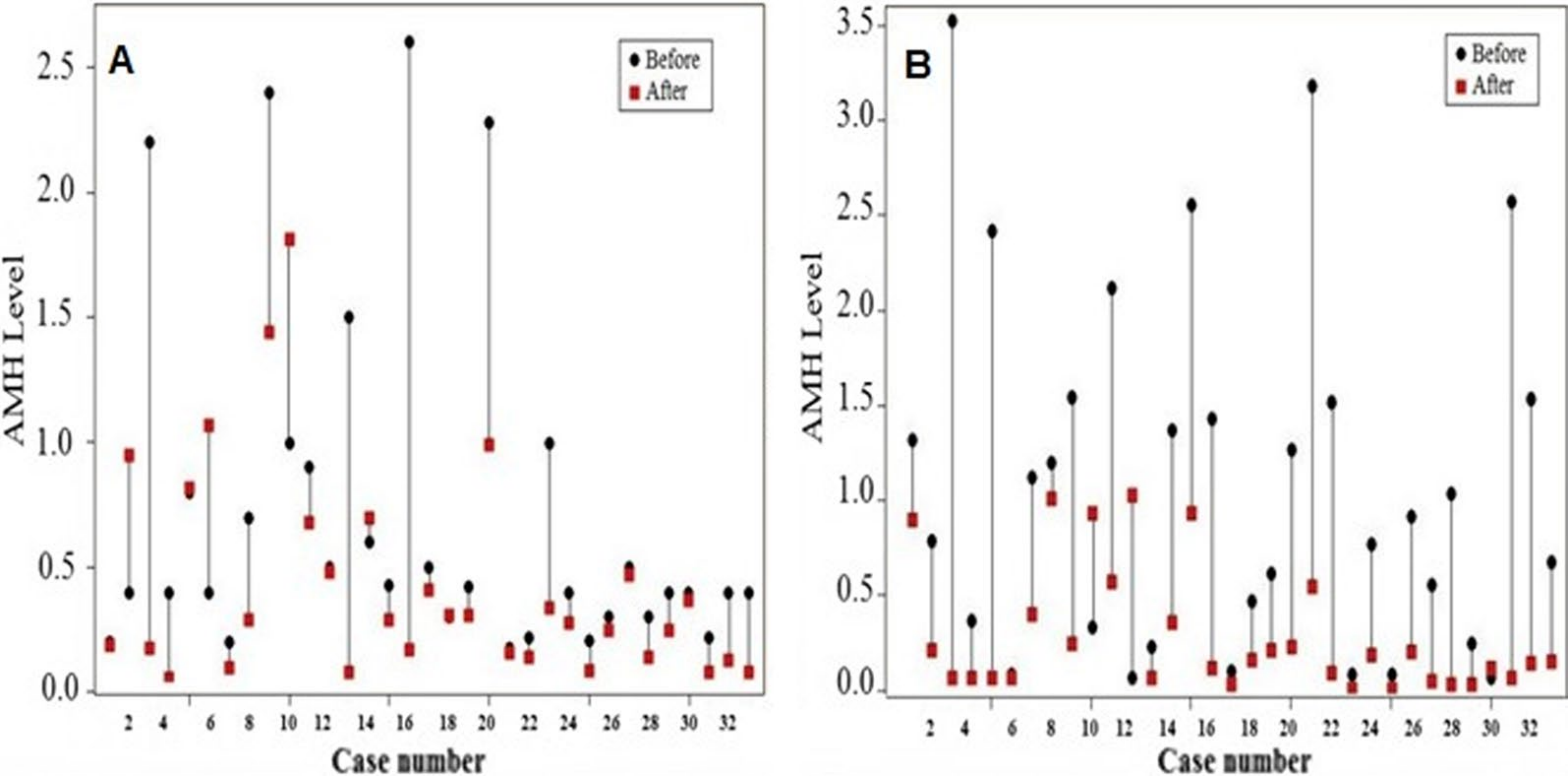
AMH values of the patients, before and after the surgery in TLH (**a**) and TAH (**b**) group

Also, the results of Wilcoxon test showed that the AMH values was significantly different before and after hysterectomy in the TLH (P = 0.001) and TAH (P < 0.001) groups (Table 2). The AMH values of all the patients, before and after the surgery were displayed in Fig. 1.

**Fig. 2:**
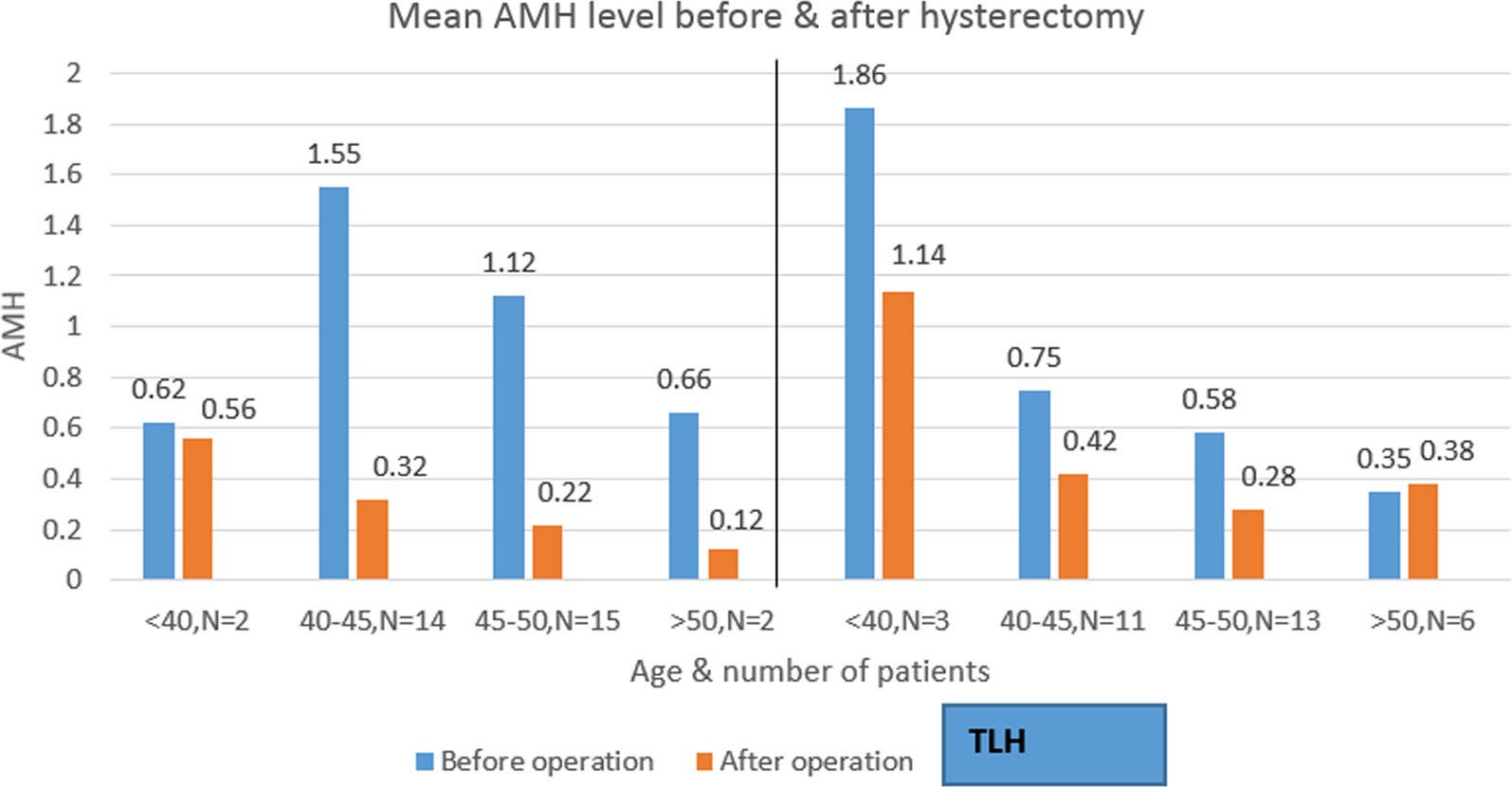
The level of AMH before and 4 months after hysterectomy

Figure 2 shows the decline in AMH based on age in both groups before and after operation.

## Discussion

The present study evaluated 33 patients in each group of laparoscopic and laparotomy hysterectomy + bilateral salpingectomy with similar baseline characteristics. Although serum levels AMH values significantly decreased below normal ranges after both methods (P = 0.003), with significant difference in decrease of post operation AMH level in laparotomy hysterectomy method. These results indicate that even after preserving ovaries, both TLH and TAH significantly decrease the ovarian reserve after 4 months. Probably, greater blood loss during TAH which lead to more use of sutures for establish hemostasis was hypothesized to decrease AMH more than laparoscopic method in our study. Therefore, it seems that the technique of hysterectomy as well as the use of electrocautery or suture can be effective on the level of AMH after surgery. However, the superiority of homeostasis techniques in maintaining ovarian reserve is still debated [25–27].

Previous studies have also evaluated the ovarian reserve after hysterectomy, although as far as the authors are concerned, none have compared AMH levels between laparoscopic and open methods, as the main objective of their study. Trabuco and colleagues compared AMH levels at baseline and 1-year follow-up between hysterectomy (n = 148) and control groups (n = 172) and showed a significantly greater decrease of AMH levels in hysterectomy group (− 40.7% vs. − 20.9%; P < 0.001) with no statistically significant difference between laparoscopic and open methods [28], indicating ovarian damage by hysterectomy, which is consistent with the results of the present study. Lee and colleagues evaluated AMH and ovarian arterial blood flow indices in 26 patients undergoing laparoscopy-assisted vaginal hysterectomy (LAVH), 6 patients undergoing TAH, and 21 age-matched controls and concluded no significant differences in these indicators after 1 week, 1 and 3 months in any of the groups [29], which is inconsistent with the results of the present study, because of lower sample size and less invasive approach (LAVH rather than TAH). Although the number of patients undergoing TAH in their study was too small for a statistically correct comparison. Moreover, they followed their patients for maximum of three months, while the current study measured the AMH after 4 months.

Other studies have principally chosen one hysterectomy method and compared pre- and post-surgical AMH values or have compared the values with a control group. Atabekoglu and colleagues compared AMH levels of 22 women undergoing TAH for uterine leiomyoma with healthy control group and reported decreased AMH after 4 months in TAH group (0.62 ± 0.9 ng/ml), which was 30% less than the control group (1.26 ± 1.78 ng/ml) (P < 0.001) [20]. The results of their study confirm the results of the present study, regarding statistically significant decrease in AMH levels after TAH (P = 0.001), although, in the present study, AMH levels at baseline and after 4 months in TAH group were much lower than the above-mentioned study that could be due to the ethnical differences and the details of the surgical techniques. In another Turkish study, Gokgozoğlu and partners showed statistically significant decrease in AMH serum levels after the first postoperative month of TAH, which resolved after three months; thus, they concluded that the decreasing effect of TAH on ovarian function is temporarily [22]. The results of their study is inconsistent with the present study, as there was a significant decrease in AMH levels after 6 months in TAH group in the present study; the reason for such a different could be the different surgical technique, as well as different bleeding volume, which was not measured in their study, as far as concerned.

Yuan compared AMH levels between two laparoscopic methods (34 patients undergoing laparoscopic supracervical hysterectomy [LSH] and 33 patients undergoing TLH) for uterine fibroids, and showed significantly decreased AMH levels 1 and 4 month(s) after surgery, compared with baseline levels (P < 0.001) [30]. The results of their study are consistent with the results of the present study, regarding statistically significant decrease in AMH levels after TLH.

Although a number of studies have concluded no significant changes in AMH levels after hysterectomy [20, 29], which might be attributable to the demographic characteristics of the study population, including age, and race [30]; although in the present study, all patient were selected from one ethnicity/race. The results of the present study, along with other studies [28, 30], suggest the devastating effect of hysterectomy on ovarian function, which is speculated to be associated with decreased ovarian blood flow [29, 31] that accelerates follicular depletion and leads to earlier menopause [13, 32]. Yet, the exact mechanism of ovarian damage following ovary-preserved hysterectomy has to be further investigated by animal studies.

The main strength of the present study is comparing TLH and TAH as the main objective of the study, for the first time, as far as the authors are concerned, with sufficient sample size. Yet, the current study had some limitations, including wide age range of participants, which could act as confounders to the results. Also, AMH was the only parameter measured that might be not a sufficient marker of ovarian function, although it is a promising predictor of ovarian function. Also, the results of the present study might be prone to selection bias, as the patients were included into the study by convenient sampling method. Considering that the study was done on the Iranian race and may not be generalizable to other races and ethnicity. Finally, it should be noted that a study that performs TAH with the electrocautery method and compare the result with this study, also seems necessary to determine the effect of surgical root on ovarian reserve.

## Conclusion

In conclusion, the comparison of serum levels of AMH between laparoscopic and laparotomy methods of hysterectomy, with no significant difference in baseline characteristics, showed significant decrease in AMH levels by both methods with significant difference between these two techniques, suggesting superiority of TLH considering ovarian reserve. Future randomized clinical trials with larger samples, as well as animal studies are required to study the pathophysiology of decreased ovarian reserve after hysterectomy, in order to be able to decline this complication.

## Data Availability

The datasets used and/or analysed during the current study are available from the corresponding author on reasonable request.

## Abbreviations

TAH: Total abdominal hysterectomy
TLH: Total laparoscopic hysterectomy
ACOG: American College of Obstetricians and Gynecologists
AMH: Anti-müllerian hormone
FSH: Follicle-stimulating hormone
E2: Estradiol
AUB: Abnormal uterine bleeding
